# Factors Associated with the Performance of a Blood-Based Interferon-γ Release Assay in Diagnosing Tuberculosis

**DOI:** 10.1371/journal.pone.0038556

**Published:** 2012-06-12

**Authors:** Sally Banfield, Elaine Pascoe, Aesen Thambiran, Aris Siafarikas, David Burgner

**Affiliations:** 1 School of Paediatrics and Child Health, University of Western Australia, Perth, Western Australia, Australia; 2 Endocrinology and Diabetes, Princess Margaret Hospital for Children, Perth, Western Australia, Australia; 3 The Institute of Health and Rehabilitation Research, University of Notre Dame, Fremantle, Western Australia, Australia; 4 The Migrant Health Unit, Perth, Western Australia, Australia; 5 School of Medicine, University of Queensland, Queensland, Australia; 6 Murdoch Childrens Research Institute, Royal Children’s Hospital, Parkville, Victoria, Australia; Hopital Raymond Poincare - Universite Versailles St. Quentin, France

## Abstract

**Background:**

Indeterminate results are a recognised limitation of interferon**-**γ release assays (IGRA) in the diagnosis of latent tuberculosis (TB) infection (LTBI) and TB disease, especially in children. We investigated whether age and common co-morbidities were associated with IGRA performance in an unselected cohort of resettled refugees.

**Methods:**

A retrospective cross-sectional study of refugees presenting for their post-resettlement health assessment during 2006 and 2007. Refugees were investigated for prevalent infectious diseases, including TB, and for common nutritional deficiencies and haematological abnormalities as part of standard clinical screening protocols. Tuberculosis screening was performed by IGRA; *QuantiFERON-TB Gold* in 2006 and *QuantiFERON-TBGold In-Tube* in 2007.

**Results:**

Complete data were available on 1130 refugees, of whom 573 (51%) were children less than 17 years and 1041 (92%) were from sub-Saharan Africa. All individuals were HIV negative. A definitive IGRA result was obtained in 1004 (89%) refugees, 264 (26%) of which were positive; 256 (97%) had LTBI and 8 (3%) had TB disease. An indeterminate IGRA result was obtained in 126 (11%) refugees (all failed positive mitogen control). In multivariate analysis, younger age (linear OR  = 0.93 [95% CI 0.91–0.95], *P*<0.001), iron deficiency anaemia (2.69 [1.51–4.80], *P* = 0.001), malaria infection (3.04 [1.51–6.09], *P* = 0.002), and helminth infection (2.26 [1.48–3.46], P<0.001), but not vitamin D deficiency or insufficiency, were associated with an indeterminate IGRA result.

**Conclusions:**

Younger age and a number of common co-morbidities are significantly and independently associated with indeterminate IGRA results in resettled predominantly African refugees.

## Introduction

*Mycobacterium tuberculosis* (MTB) infects over one third of the global population and tuberculosis (TB) disease results in approximately 1.3 million deaths annually, largely in high incidence, resource-poor countries. [1] With increasing migration from high to low incidence regions, the majority of TB notifications in resource-rich countries such as Australia [2] and the UK [3] are in those born overseas. Active TB disease results either from reactivation of latent TB infection (LTBI), or from uncontrolled primary infection, particularly in younger children, who usually acquire infection from an infectious adult. [4] The greatest risk of progression from TB infection to disease is in the post-migration period. Identification and treatment of those with asymptomatic LTBI is therefore an important component of the initial health assessment of refugees and others from high incidence regions. Coordinated TB screening of high risk individuals is integral to national TB control policies in resource-rich countries, [5], [6] and reduces the overall transmission rate of TB. [7], [8], [9].

Given the delay and difficulties in reaching a microbiological diagnosis of TB disease, especially in children, and the lack of positive microbiology in LTBI, the diagnosis of TB infection is mostly reliant on the demonstration of immunological memory to mycobacterial antigens. The tuberculin skin test (TST) remains widely used for this purpose, despite well-documented limitations, especially in relation to specificity. [10] Interferon-γ release assays (IGRA), which quantify the interferon (IFN)-γ response of whole blood (*QuantiFERON®-TB Gold*, QFT and *QuantiFERON®-TB Gold In-Tube*, QFT-IT, Cellestis, Australia) or isolated mononuclear cells (T-Spot.*TB*, Immunotech, UK) to largely MTB-specific antigens, are increasingly used in resource-rich settings. [11] These assays have greater specificity than TST, but their role as screening tools for LTBI remains poorly defined. Indeterminate results, which are reported in up to 35% of QFT [12], [13] and 15% of QFT-IT assays [14] are a significant potential limitation to their utility, particularly in young and/or immunocompromised patients. [12], [15], [16].

Immune responses to TB are influenced by a variety of factors, including younger age, vitamin D insufficiency or deficiency, and concurrent infections, particularly malaria, helminth and viral infections. [17], [18], [19] The effect of iron deficiency on TB responses are ill-defined, but iron deficiency impairs cellular immune responses and increases susceptibility to infection more generally. [20] There are limited data on whether these factors affect the performance of IGRA.

We investigated the effect of these co-morbidities on the performance of QFT and QFT-IT as a screening tool for TB infection in a large cohort of recently resettled unselected paediatric and adult refugees in Australia.

## Methods

### Study Population and Design

Ethical approval was obtained from the Princess Margaret Hospital for Children Ethics Committee, Perth, Western Australia, Australia (*Governance Evidence Knowledge Outcomes* approval). This was a retrospective cross-sectional study of unselected consecutive newly arrived refugees, who had an initial health assessment at a designated refugee health assessment centre (Perth, Western Australia, Australia) between 1st January 2006 and 31st December 2007. The study population included 265 children (of a total of 573 refugee children included, 46.2%) who were previously enrolled in a reported comparative study of QFT-IT, T-SPOT.*TB* and TST in Asian and African refugee children, for which experimental blood sampling was only performed once routine screening investigations had been completed. [14] The current study was a larger and more detailed assessment of IGRA performance (both QFT and QFT-IT), with additional paediatric subjects and the inclusion of adults, as well as analysis of additional common co-morbid conditions.

The initial refugee health assessment is performed over two visits, with routine information collected on country of birth and of refuge, seasonality of health assessment, and body mass index. [21] It was not possible to obtain an accurate TB contact history, as many refugees previously lived in over-crowded refugee camps, [22] and the usual parameters for defining ‘household contact’ were difficult to ascertain.

Laboratory investigations included an immunochromatographic assay for *Plasmodium falciparum* (Binax NOW®, Portland, USA), single thick and thin blood films for malaria, serology for schistosomiasis (enzyme immunoassay, EIA or indirect haemagglutination assay, IHA) and for strongyloidiasis (EIA), [21] automated full blood count and leucocyte differential, iron stores (iron, transferrin, transferrin saturation and ferritin), total calcium, phosphate, alkaline phosphatase and 25-hydroxyvitamin D, all performed by standard laboratory assays. Analysis of stool specimens for faecal parasites is not part of the routine health assessment in Western Australia and approximately 80% of refugees received empiric anti-helminth treatment shortly before migration.

### Interferon-γ Release Assays

All subjects had an IGRA (QFT during 2006 and QFT-IT from 2007 onwards). The Western Australian TB Control Program replaced TST with IGRA as the preferred TB screening method, partly because of logistical difficulties in accessing recently resettled and often mobile refugee patients 48–72 h post-TST. [14] A single IGRA was performed on each patient at the initial health screening in accordance with the manufacturer’s instructions. Both QFT and QFT-IT use phytohaemagluttinin (PHA) as the positive mitogen control and have an antigen-free negative (nil) control. In the QFT, responses to MTB-specific antigens CFP-10 and ESAT-6 are assayed in two separate tubes, whereas the QFT-IT uses a single tube containing the antigens CFP-10, ESAT-6 and TB7.7. Interpretation of the IGRA result was according to the manufacturer’s guidelines. A positive IGRA result was defined as an IFN-γ response to one or more MTB-specific antigens above the recommended cut-off, irrespective of the IFN-γ response to the mitogen control. A negative IGRA result was an IFN-γ response below cut-off for all MTB-specific proteins, with a response to the mitogen control above cut-off. An indeterminate result was defined as either an IFN-γ response below the cut-off for both MTB-specific proteins and the mitogen control, or an IFN-γ response above the cut-off in the nil control.

### Haematological analyses

Anaemia was defined as a concentration of haemoglobin less than age and gender-adjusted values used in the Western Australian population (PathWest Laboratories, Perth, Western Australia). Iron deficiency was defined by at least two abnormal iron parameters (iron, ferritin, transferrin and transferrin saturation, corrected for age and gender norms). [23] Iron deficiency anaemia was defined as coexisting iron deficiency and anaemia. The serum 25-hydroxyvitamin D levels were classified according to international standards of deficient (<27.5 nmol/L), insufficient (27.5–78 nmol/L) and sufficient (>78 nmol/L). [24].

### Diagnosis of Infectious Diseases

Helminth infection was identified by a positive serology for schistosomiasis (EIA>1.2 and/or IHA>16), and/or positive serology for strongyloides (EIA>0.45), and/or a peripheral eosinophil count of ≥0.7×10^9^/L. Equivocal serology for either schistosomiasis or strongyloides was not considered indicative of infection if the eosinophil count was below the diagnostic cut-off. Malaria infection was diagnosed by either a positive blood film and/or a positive immunochromatographic test for *P. falciparum*.

### Statistical Analysis

Analyses were performed using data from patients with no missing clinical, demographic or laboratory data. Univariate logistic regression models were used to obtain unadjusted odds ratios for all characteristics potentially associated with a positive (versus negative) or an indeterminate (versus definite positive or negative) IGRA result. Age was centred at the median to allow inclusion of a quadratic effect for this characteristic. The models for malaria, helminth infection, iron deficiency and iron deficiency anaemia were additionally assessed using characteristic*age interaction effects to look for possible modification of the effect of each characteristic by age. Multivariate logistic regression models were used to identify characteristics that were independently associated with a IGRA positive result and, separately, to identify factors independently associated with an indeterminate result. All characteristics were provisionally included in the multivariate models and backward elimination was used to remove characteristics sequentially. At each step, the characteristic with the largest multivariate *P*-value was removed. The final models included only those characteristics with multivariate *P*<0.05. Multiple imputation was used to ascertain whether exclusion of individuals from analyses, due to incomplete data, may have introduced bias in the logistic regression results. For each outcome, 50 datasets were imputed and analysed with multivariate logistic regression with the 50 sets of regression results combined using Rubin’s rules. [25] All analyses were performed using Stata version 10.1 (Stata Corporation, College Station, TX).

## Results

### Patient Characteristics

A total of 1760 refugees were assessed during the study period and complete data were available on 1130 (64%). There were 573 (51%) children (<17 years) and 557 (49%) adults. Just under half (49%) were male. The majority of refugees (1041, 92%) were from sub-Saharan Africa, with the remainder from Asia, predominantly Myanmar (Table 1).

Of the 1130 refugees, 438 (39%) had a QFT and 692 (61%) had a QFT-IT. A definitive (non-indeterminate) IGRA result was obtained in 1004 (of 1130, 89%), of which 264 (26%) were positive. Of those with a positive IGRA, eight (3%) had TB disease, which was diagnosed by clinical, radiological and/or microbiological parameters by experienced physicians. Of the 126 (11%) refugees with an indeterminate IGRA result, all were the result of a failed positive mitogen control. Malaria infection was present in 44 (4%) individuals and a helminth infection in 444 (39%), of which the majority were schistosomiasis. Iron deficiency was present in 166 (15%) and iron deficiency anaemia in approximately half of these (7%). Over 85% of the cohort was either vitamin D insufficient (79%) or deficient (7%) (Table 2).

**Table 1:** Demographic characteristics of study population (n = 1130).

Characteristic	Estimate*
Age (years)	19.8±13.4
Gender: male (%)	552 (48.8%)
*Country of Birth* (%)	
Africa	1041 (92.1%)
Sudan	318 (28.1%)
Congo	140 (12.4%)
Liberia	113 (10.0%)
Burundi	78 (6.9%)
Tanzania	58 (5.1%)
Sierra Leonne	57 (5.0%)
Africa: other**	277 (24.5%)
Asia	88 (7.8%)
Burma	68 (6.0%)
Asia: Other+	20 (1.8%)
Other++	1 (0.1%)
*Seasonality of MHU visit* (%)	
Summer	156 (13.8%)
Autumn	239 (21.1%)
Winter	355 (31.4%)
Spring	380 (33.6%)
Body mass index (kg/m^2^)	20.3±4.7
MMR vaccine received (%)	830 (73.4%)

* Estimate values represent mean ± standard deviation or median (interquartile range) or frequency (percent) of study population.

** Other represents 17 countries of birth in Africa with <4% of study population.

+ Other represents 2 countries of birth in Asia with <4% of study population.

++ Other represents 1 country of birth outside of Asia or Africa with <4% of study population.

**Table 2:** Clinical characteristics of study population (n = 1130).

Characteristic	Estimate*
*IGRA result* ** *(%*)	
Positive	264 (23.4% of all IGRA results)
Positive: *≥17 years*	*207 (78.4% of positive IGRA results*)
Positive: *<17 years*	*57 (21.6% of positive IGRA results*)
Negative	740 (65.5% of all IGRA results)
Negative: *≥17 years*	*317 (42.8% of negative IGRA results*)
Negative: *<17 years*	*423 (57.2% of negative IGRA results*)
Indeterminate	126 (11.1% of all IGRA results)
Indeterminate: *≥17 years*	*33 (26.2% of indeterminate IGRA results)*
Indeterminate: *<17 years*	*93 (73.8% of indeterminate IGRA results)*
*Malaria infection (%)*	
Yes	44 (3.9%)
No	1086 (96.1%)
*Schistosoma* *serology (%)*	
Positive	298 (27.7%)
Negative	744 (69.3%)
Equivocal	32 (3.0%)
*Strongyloides* *serology (%)*	
Positive	72 (6.4%)
Negative	1023 (90.7%)
Equivocal	33 (2.9%)
*Helminth* *infection* + *(%)*	
Yes	444 (39.3%)
No	686 (60.7%)
*Vitamin D level* ++ *(%)*	
Sufficient	161 (14.2%)
Insufficient	891 (78.9%)
Deficient	78 (6.9%)
Haemoglobin (g/L)	130.5±17.6
Iron (µmol/L)	13.9±5.8
Transferrin (µmol/L)	36.7±5.7
Transferrin saturation (%)	19.7±9.0
Ferritin (µg/L)	44 (24–78)
Anaemia (%)	203 (18.0%)
Iron Deficiency (%)	166 (14.7%)
Iron deficiency anaemia (%)	80 (7.1%)
Alkaline phosphatase (μ/L)	138.5 (75–284)
Phosphate (mmol/L)	1.4 (1.2–1.7)
Total calcium (mmol/L)	2.4±0.1
Eosinophil count (×10^9^/L)	0.4±0.6

* Estimate values represent mean ± standard deviation or median (interquartile range) or frequency (percent) of study population.

** Screening through only the QuantiFERON®-TB Gold or QuantiFERON®-TB Gold In-tube assays.

+ Defined as a positive serology to either *Schistosoma* or *S. stercoralis*, or an eosinophil count >0.7×10^9^/L.

++ Defined as deficient (<27.5 nmol/L), insufficient (27.5–78 nmol/L) and sufficient (>78 nmol/L).

### Characteristics Associated with a Positive IGRA Result

**Figure 1:**
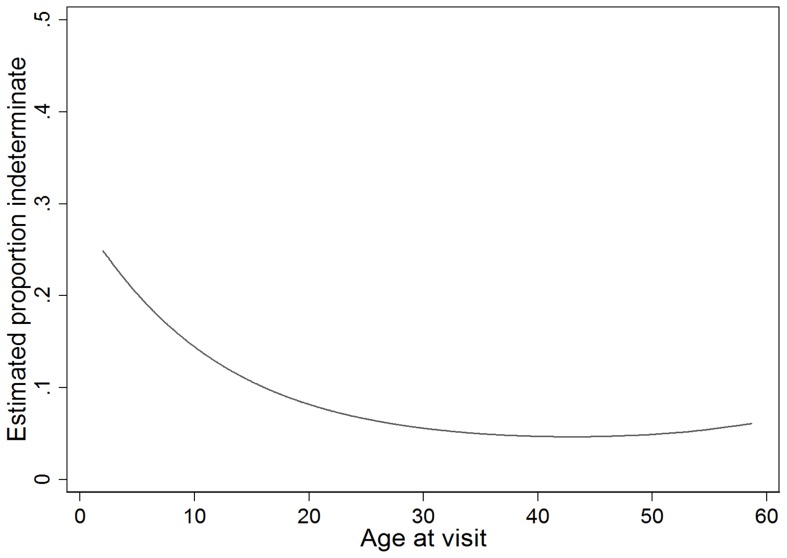
The probability of a positive IGRA based on age. This figure shows the estimated proportion of positive IGRA results, compared to negative IGRA results, depending on the age at assessment. *Not adjusted for other covariates in the multivariate model.*

In the univariate analysis, older age (linear odds ratio 1.10, 95% confidence interval (CI) 1.08–1.12, *P*<0.001) and a health assessment performed in summer (*P* = 0.022, compared to other seasons) were independently associated with positive IGRA. Vitamin D status was not associated with a positive IGRA result on univariate analysis and was therefore not carried forward into the multivariate model (Table 3). There was a strong positive linear association between age and the odds of a positive IGRA result until approximately 30 years of age, beyond which the association declined (Figure 1). The modest reversal in this trend from approximately 45 years of age onwards was not significant (cubic effect coefficient for age *P* = 0.64).

**Table 3:** Characteristics associated with a positive IGRA result (n = 1004).

Variable	Univariate odds ratio (95% CI*)	P value	Multivariate odds ratio (95% CI*)+	P value+
*Gender*		0.189		–
Male	1			
Female	0.83 (0.62–1.10)			
*Age at visit*		<0.001		<0.001
Linear	1.10 (1.08–1.12)		1.10 (1.08–1.12)	
Quadratic	1.00 (1.00–1.00)		1.00 (1.00–1.00)	
*Country of birth*		0.287		–
Not Africa	1			
Africa	1.35 (0.78–2.35)			
*Seasonality of visit*		0.006		0.022
Summer	1		1	
Autumn	0.46 (0.28–0.74)		0.49 (0.29–0.82)	
Winter	0.52 (0.34–0.79)		0.52 (0.33–0.83)	
Spring	0.64 (0.42–0.98)		0.60 (0.39–0.95)	
*Vitamin D*		0.431		–
Deficient	1			
Insufficient	1.28 (0.72–2.28)			
Sufficient	1.53 (0.79–2.95)			
Body mass index++	1.04 (0.92–1.18)	0.531		–
Helminth infection	1.84 (1.39–2.45)	<0.001		–
Malaria infection	1.42 (0.65–3.07)	0.376		–
Iron deficiency	0.92 (0.61–1.40)	0.712		–
Iron deficiency anaemia	0.83 (0.45–1.53)	0.541		–
Eosinophil count (Log(×10^9^/L)	1.08 (0.95–1.22)	0.249		–

* CI = confidence interval.

+ Multivariate odds ratio and p-value reported if significant (*P*<0.05).

++ Age- and gender-adjusted variable.

### Characteristics Associated with an Indeterminate IGRA Result

**Figure 2:**
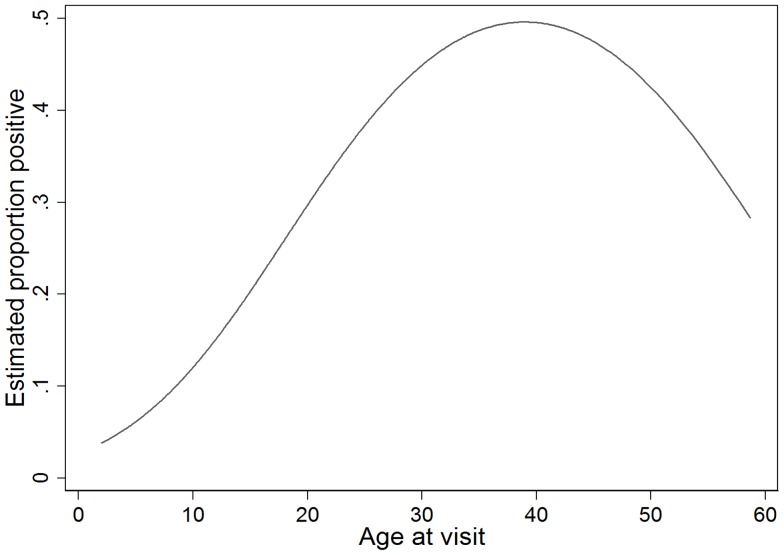
The probability of an indeterminate IGRA based on age. This figure shows the estimated proportion of indeterminate IGRA results depending on the age at the MHU visit. *Not adjusted for other covariates in the multivariate model.*

Younger age, helminth infection, malaria infection, and iron deficient anaemia were significantly and independently associated with an indeterminate IGRA result (Table 4). The odds of an indeterminate IGRA result decreased linearly with increasing age (linear odds ratio (OR) 0.93, 95% CI 0.91–0.95, *P*<0.001). At approximately 15 years of age this association gradually declined with no evidence of an association between age and indeterminate IGRA beyond 25–35 years (Figure 2). Helminth infection (OR 2.26, 95% CI 1.48–3.46, *P*<0.001), malaria infection (OR 3.04, 95% CI 1.51–6.09, *P* = 0.002), and iron deficient anaemia (OR 2.69, 95% CI 1.51–4.80, *P* = 0.001) all significantly increased the odds of an indeterminate IGRA result. Vitamin D insufficiency or deficiency had no effect on the likelihood of an indeterminate IGRA result. Multivariate logistic regression results derived from multiple imputation for characteristics associated with a positive IGRA or indeterminate IGRA differed little from results derived from refugees with complete data (data not shown).

The associations between an indeterminate IGRA and malaria infection, helminth infection, iron deficiency and iron deficiency anaemia were not modified by age; the interaction effects added to the relevant univariate models were all non-significant (malaria*age *P* = 0.153; helminth infection*age *P* = 0.497; iron deficiency*age *P* = 0.237; and iron deficient anaemia*age *P* = 0.460).

**Table 4:** Characteristics associated with an indeterminate IGRA result (n = 1130).

Variable	Univariate odds ratio (95% CI*)	*P* value	Multivariate odds ratio (95% CI*)+	*P* value+
*Gender*		0.932		–
Male	1			
Female	0.98 (0.68–1.42)			
*Age at visit*		<0.001		<0.001
Linear	0.94 (0.93–0.96)		0.93 (0.91–0.95)	
Quadratic	1.00 (1.00–1.00)		1.00 (1.00–1.00)	
*Country of birth*		0.746		–
Not Africa	1			
Africa	1.12 (0.55–2.30)			
*Seasonality of visit*		0.125		–
Summer	1			
Autumn	1.60 (0.86–2.99)			
Winter	0.93 (0.49–1.73)			
Spring	1.00 (0.54–1.85)			
*Vitamin D*		0.182		–
Deficient	1			
Insufficient	2.50 (0.89–6.97)			
Sufficient	2.04 (0.66–6.32)			
Body mass index++	0.97 (0.82–1.14)	0.701		–
Helminth infection	1.42 (0.98–2.05)	0.067	2.26 (1.48–3.46)	<0.001
Malaria infection	4.06 (2.09–7.88)	<0.001	3.04 (1.51–6.09)	0.002
Iron deficiency	1.99 (1.27–3.12)	0.003		–
Iron deficiency anaemia	2.74 (1.58–4.77)	<0.001	2.69 (1.51–4.80)	0.001
Eosinophil (Log(×10^9^/L)	1.16 (0.99–1.37)	0.066		–

* CI = confidence interval.

+ Multivariate odds ratio and p value reported if significant (*P*<0.05).

++ Age- and gender-adjusted variable.

## Discussion

In this large retrospective study of recently resettled predominantly African child and adult refugees, more than a quarter had a positive IGRA, the majority due to LTBI. This is comparable to data reported from paediatric [14], [26], [27] and adult refugees [28] resettled in Australia and other resource-rich countries. One in eight individuals had an indeterminate IGRA result, all due to failure of the positive mitogen control. An indeterminate result complicates management and increases costs, as further TB diagnostic testing (by TST and/or an alternative IGRA) and other investigations are often necessary. Younger age, concurrent malaria or helminth infection, iron deficiency and iron deficiency anaemia were significantly and independently associated with an indeterminate IGRA result. In a previous study that included a sub-set of children from the current cohort, we found that helminth infection, and either malaria or viral hepatitis infection, increased the likelihood of an indeterminate QFT-IT. [14] The current study confirms and extends these findings. The inclusion of a large adult cohort, together with interaction analysis for age-modifying effects, none of which were statistically significant, indicates that these are not age-specific associations.

The aetiology of the failed positive mitogen control responsible for the indeterminate IGRA results is likely to be multi-factorial. T cell production of IFN-γ, a prototypical T-helper 1 (Th1) cytokine is relatively deficient in younger children, [29] partly due to increased methylation of the IFN-γ promoter. [30] Helminth infection induces a Th2 immune phenotype, with concomitant reduced Th1 response and increased regulatory T-cells, which reduces overall IFN-γ production. [31] In a small study of Indian adult patients with LTBI, concurrent filarial infection, which is known to suppress Th1 and enhance Th2 responses, was associated with down regulation of IFN-γ and enhanced interleukin (IL)-4 responses to mycobacterial antigens *in vitro*. [32] Although screening for filarial infection is not part of standard care in refugees resettled in Australia, the immunological mechanisms may be similar to those in helminth infection and both filarial and helminth infections suppress responses to BCG vaccination. [33].

Malaria was also associated with increased risk of an indeterminate IGRA result. Despite the considerable epidemiological overlap between these two major causes of global mortality, there are few data on the interaction between malaria and immune responses to MTB. In the resettled refugee population, many malarial infections are chronic, with low level parasitaemia and minimal symptoms. [34], [35] Both acute and chronic asymptomatic malaria infection are broadly immunosuppressive, increasing susceptibility to infection with other pathogens such as salmonella and reducing responses to polysaccharide vaccines. [36] Similar mechanisms may contribute to reduced mitogen responses in IGRA.

The lack of an association between reduced vitamin D levels and IGRA performance was an unexpected finding. Low levels of vitamin D are present in the majority of resettled refugees in Australia. [22] Vitamin D is a key modulator of the complex immune responses to TB infection [37] and was used as an anti-TB agent in the pre-antibiotic era. [38] Reduced vitamin D levels are associated with increased risk of progression from LTBI to TB disease in Indian adults recently resettled in the UK. [39] *In vitro* responses to mycobacterial antigens are associated with functional polymorphisms of the vitamin D binding protein, an effect that is only observed in those who were also vitamin D deficient. [40] Vitamin D supplementation of *in vitro* human T cells selectively reduces mitogen responses and IL-2 production, possibly due to the lack of accessory cells. [41] A single dose of vitamin D supplementation given to adults with LTBI increased their IFN-γ responses to mycobacterial antigens. [42] It is unclear why low levels of vitamin D did not affect the IGRA responses in the current study.

The association between a positive IGRA result and summer months was not mediated by differences in vitamin D levels. Refugees are often resettled in large groups from specific countries of refuge, such as a single refugee camp. Thus the seasonal association may reflect increased local prevalence of TB infection in a specific group of refugees who were resettled *en masse* in late spring in early summer (and therefore had an IGRA performed during the summer months), although this is difficult to substantiate.

The strengths of the current study include the size of the unselected cohort, which included both children and adults and which was representative of the resettling refugee population in Australia during the study period. All participants were investigated using standardised and extensive investigations. Limitations include the fact that the majority of the cohort was from sub-Saharan Africa and there was insufficient representation of other ethnic groups to draw firm conclusions about the generalisability of these data. Although the IGRA used changed from QFT to QFT-IT during the study period, this did not alter the findings (data not shown). A further limitation is the lack of concurrent TST data to ascertain if the co-morbidities affected the performance of TST in an analogous way. As the diagnosis of co-morbidities was determined by clinical guidelines that aimed to identify important infections in a cost-effective way, we cannot comment on the possible effects of other potentially prevalent infections. Approximately 80% of the refugee intake into Australia has received empiric anti-helminth treatment prior to departure and routine stool microscopy is not performed in Western Australia. We are therefore unable to comment on the possible modifying effects of anti-helminth treatment, nor on the differential effects of other helminths, which may also have significant immunomodulatory properties. [43] Prospective studies, including those in other ethnic groups that repeat the IGRA following treatment of these co-morbidities and those that investigate *ex-vivo* cellular immune function are warranted. These data would address whether the observed associations are causal and identify underlying mechanisms.

In conclusion the current findings indicate that indeterminate IGRA results occurred in a substantial proportion of African paediatric and adult refugees, potentially limiting the utility of this investigation as a screening tool for TB infection. Co-morbidities including chronic infections and nutritional deficiencies are independently associated with an increased risk of an indeterminate IGRA result.
